# Tracking the Evolutionary Trends Among Small-Size Fishes of the Genus *Pyrrhulina* (Characiforme, Lebiasinidae): New Insights From a Molecular Cytogenetic Perspective

**DOI:** 10.3389/fgene.2021.769984

**Published:** 2021-10-06

**Authors:** Renata Luiza Rosa de Moraes, Francisco de Menezes Cavalcante Sassi, Luiz Antonio Carlos Bertollo, Manoela Maria Ferreira Marinho, Patrik Ferreira Viana, Eliana Feldberg, Vanessa Cristina Sales Oliveira, Geize Aparecida Deon, Ahmed B. H. Al-Rikabi, Thomas Liehr, Marcelo de Bello Cioffi

**Affiliations:** ^1^ Laboratorio de Citogenética de Peixes, Departamento de Genética e Evolução, Universidade Federal de São Carlos (UFSCar), São Carlos, Brazil; ^2^ Museu de Zoologia da Universidade de São Paulo (MZUSP), São Paulo, Brazil; ^3^ Laboratório de Sistemática e Morfologia de Peixes, Departamento de Sistemática e Ecologia (DSE), Universidade Federal da Paraíba (UFPB), João Pessoa, Brazil; ^4^ Laboratório de Gentética Animal, Instituto Nacional de Pesquisa da Amazônia, Coordenação de Biodiversidade, Manaus, Brazil; ^5^ Laboratório de Biologia Cromossômica, Estrutura e Função, Departamento de Biologia Estrutural, Molecular e Genética, Universidade Estadual de Ponta Grossa, Ponta Grossa, Brazil; ^6^ Institute of Human Genetics, University Hospital Jena, Jena, Germany

**Keywords:** fishes, repetitive DNAs, karyotype evolution, sex chromosomes, evolution

## Abstract

Miniature fishes have always been a challenge for cytogenetic studies due to the difficulty in obtaining chromosomal preparations, making them virtually unexplored. An example of this scenario relies on members of the family Lebiasinidae which include miniature to medium-sized, poorly known species, until very recently. The present study is part of undergoing major cytogenetic advances seeking to elucidate the evolutionary history of lebiasinids. Aiming to examine the karyotype diversification more deeply in *Pyrrhulina*, here we combined classical and molecular cytogenetic analyses, including Giemsa staining, C-banding, repetitive DNA mapping, comparative genomic hybridization (CGH), and whole chromosome painting (WCP) to perform the first analyses in five *Pyrrhulina* species (*Pyrrhulina* aff. *marilynae*, *Pyrrhulina* sp., *P. obermulleri*, *P. marilynae* and *Pyrrhulina* cf. *laeta*). The diploid number (2n) ranged from 40 to 42 chromosomes among all analyzed species, but *P. marilynae* is strikingly differentiated by having 2n = 32 chromosomes and a karyotype composed of large meta/submetacentric chromosomes, whose plesiomorphic status is discussed. The distribution of microsatellites does not markedly differ among species, but the number and position of the rDNA sites underwent significant changes among them. Interspecific comparative genome hybridization (CGH) found a moderate divergence in the repetitive DNA content among the species’ genomes. Noteworthy, the WCP reinforced our previous hypothesis on the origin of the X_1_X_2_Y multiple sex chromosome system in *P. semifasciata*. In summary, our data suggest that the karyotype differentiation in *Pyrrhulina* has been driven by major structural rearrangements, accompanied by high dynamics of repetitive DNAs.

## Introduction

Characiformes comprise a very diverse and abundant freshwater order (Nelson et al., 2016), in which the family Lebiasinidae is represented by 75 valid species (Fricke et al., 2021) widely distributed across South and Central America (Weitzman and Weitzman, 2003). The phylogenetic relationships of the Lebiasinidae remained in doubt for a long time, but more recent phylogenetic analysis indicate their proximity to the Ctenoluciidae (Calcagnotto et al., 2005; Oliveira et al., 2011), which was also reinforced by the different studies (Arcila et al., 2017; Betancur-R et al., 2019; Melo et al., 2021). Most Lebiasinidae species reach about 60 mm of Standard Length (SL), but miniature species, not surpassing a maximum of 26 mm SL, is found within the Pyrrhulininae, whereas medium-sized species up to 150 mm SL can be found within Lebiasininae (Weitzman and Weitzman, 2003).

Because of their small sizes and difficulties in obtaining good chromosomal preparations, species of Lebiasinidae were, for a long time, little analyzed in terms of cytogenetics, with scarce references mainly on the chromosomal number of few species (Scheel, 1973; Oliveira et al., 1991; Arai, 2011). However, this scenario has recently undergone significant changes with the methodological advance of cytogenetics and its applicability among small to miniature fishes of *Pyrrhulina*, *Lebiasina*, *Copeina*, and *Nannostomus* genus (de Moraes et al., 2017, de Moraes et al.,2019; Sassi et al., 2019; Toma et al., 2019; Sassi et al., 2020; Sember et al., 2020).

*Pyrrhulina* is one of the most speciose genera of the subfamily Pyrrhulininae, with 19 valid small species (Fricke et al., 2021), ranging from 30.4 to 85 mm SL (Weitzman and Weitzman, 2003; Netto-Ferreira and Marinho, 2013). The genus is among the most problematic, with many poorly known species, species complexes, and old taxonomic problems (Netto-Ferreira and Marinho, 2013). The first *Pyrrhulina* species to have some chromosomal data evidenced was *Pyrrhulina* cf. *australis*, with 2n = 40 chromosomes, mainly acrocentric ones (Oliveira et al., 1991). Taxonomic boundaries of *P. australis* are still poorly defined, demonstrated in subsequent studies (de Moraes et al., 2017; de Moraes et al.,2019) of two morphotypes. Both *P. australis* and *Pyrrhulina* aff. *australis* showed similar data 2n = 40 (4st + 36a), distinct from *P. brevis*, 2n = 42 (2sm + 4st + 36a), with no evidence of heteromorphic sex chromosomes in the three species (de Moraes et al., 2017; de Moraes et al., 2019). Another species, *P. semifasciata*, was analyzed, presenting 2n = 42 (4st + 38a) in females, and 2n = 41 (1m + 4st + 36a) in males, the latter with three unpaired chromosomes because of a multiple X_1_X_1_X_2_X_2_/X_1_X_2_Y sex chromosome system (de Moraes et al., 2019). This occurrence was also confirmed by comparative genomic hybridizations (CGH) and whole-chromosome painting (WCP), with some indications that the Y chromosome originated by centric fusions of non-homologous acrocentric chromosomes (de Moraes et al., 2019).

To improve the knowledge of the evolutionary processes within the genus *Pyrrhulina*, we combined classical and molecular cytogenetic analyses, including Giemsa staining, C-banding, repetitive DNA mapping, comparative genomic hybridization (CGH), and whole chromosome painting (WCP to perform the first analyses in five *Pyrrhulina* species (*Pyrrhulina* aff. *marilynae*, *Pyrrhulina* sp., *P. obermulleri*, *P. marilynae* and *Pyrrhulina* cf. *laeta*). The results highlighted relationships and particular evolutionary paths at the chromosomal and genomic levels among the species. In addition, the hypothesis on the origin of the multiple sex chromosome system in *P. semifasciata* is validated.

## Materials and Methods

### Animals

**FIGURE 1:**
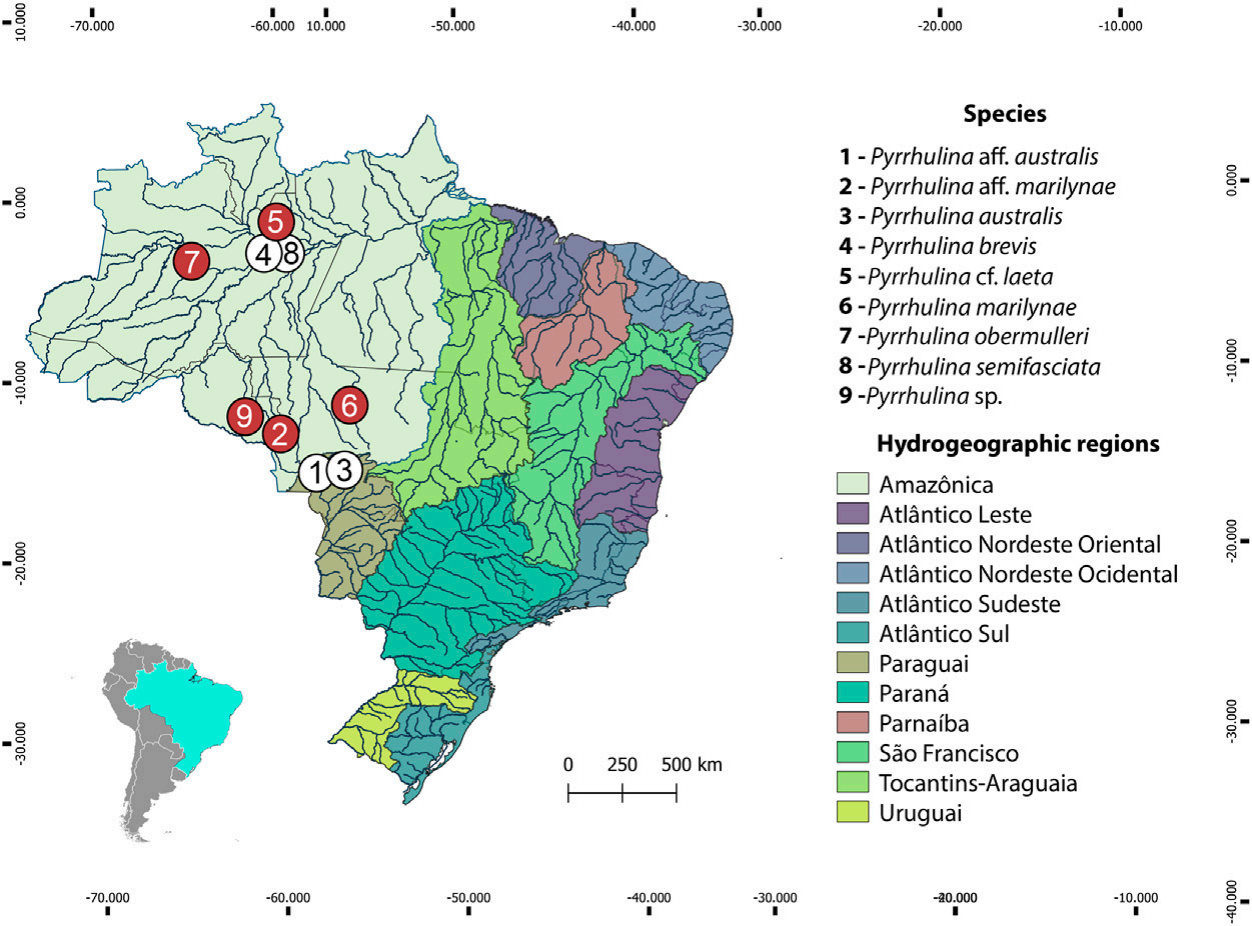
Brazilian collection sites of the *Pyrrhulina* species cytogenetically investigated in the present study (red circles) and the ones previously cytogenetically analyzed (white circles: data from (de Moraes et al., 2017; de Moraes et al., 2019).

The collection sites, number, and sex of the specimens investigated are presented in Figure 1, Table 1. Part of the sampling (Figure 1, white circles) resembles the one previously analyzed by de Moraes et al. (2017), de Moraes et al. (2019) with different cytogenetic and molecular methods. Animals were collected with the authorization of the Brazilian environmental agency ICMBIO/SISBIO (license no. 48628-14) and SISGEN (A96FF09). All species were properly identified by morphological criteria, and the specimens were deposited in the fish collection of the Museu de Zoologia da Universidade de São Paulo (MZUSP) under the voucher numbers (119077, 119079, 123073, 123080) and the Universidade Federal da Paraíba (UFPB) museum under the voucher number (12079, 12080, 12082 and 12083). Experiments followed ethical and anesthesia conducts and were approved by the Ethics Committee on Animal Experimentation of the Universidade Federal de São Carlos (process number CEUA 1853260315).

**TABLE 1:** Geographical coordinates, sample size, and diploid number of *Pyrrhulina* (Characiformes, Lebiasinidae) species collected in Brazil.

Species	Locality	Sample size	2n (Sex)	References
*Pyrrhulina* aff. *australis*	Rio Sepotuba, Lambari D’Oeste—MT (15°11′28.0″S 57°41′30.7″W)	16♂ 22♀	40♂♀	de Moraes et al. (2017)
*Pyrrhulina* aff. *marilynae*	Igarapé 12 de Outubro, Comodoro—MT (12°58′41.0″S 60°00′34.0″W)	14♂ 10♀	40♂♀	This study
*P. australis*	Barra do Bugres—MT (15°04′27.5″S 57°11′05.4″W)	18♂ 30♀	40♂♀	de Moraes et al. (2017)
*P. brevis*	Reserva Florestal Adolpho Ducke, Manaus—AM (2°58′20.7″S 59°55′53.0″W)	17♂ 13♀	42♂♀	de Moraes et al. (2019)
*Pyrrhulina* cf. *laeta*	Presidente Figueiredo—AM (1°59′10.8″S 60°03′40.8″W)	07♂ 05♀	42♂♀	This study
*P. marilynae*	Ipiranga do Norte—MT (11°36′02.0″S 55°56′27.0″W)	14♂ 08♀	32♂♀	This study
*P. obermulleri*	Tefé—AM (3°25′50.7″S 64°44′54.8″W)	21♂ 12♀	42♂♀	This study
*P. semifasciata*	Careiro—AM (3°51′00.0″S 60°04′00.0″W)	12♂ 09♀	41♂42♀	de Moraes et al. (2019)
*Pyrrhulina* sp	Represa, Alto Alegre dos Parecis—RO (12°11′58.0″S 61°46′47.7″W)	19♂ 29♀	40♂♀	This study

### Chromosomal Preparations and Analysis of the Constitutive Heterochromatin

Mitotic chromosomes were obtained from kidney cells by the protocol described in Bertollo et al. (2015). The distribution of constitutive heterochromatin was observed by the C-banding methodaccording to (Sumner, 1972).

### Repetitive DNA Mapping with Fluorescence *in situ* Hybridization (FISH)

The 5S rDNA probe included 120 base pairs (bp) of the 5S rDNA gene coding region and 200 bp of non-transcribed spacer (NTS) (Pendás et al., 1994). The 18S rDNA probe was composed of a 1,400-bp-long segment of the 18S rDNA coding region (Cioffi et al., 2009). Both probes were directly labeled with the Nick-Translation Mix Kit (Jena Bioscience, Jena, Germany)—18S rDNA with ATTO488-dUTP and 5S rDNA with ATTO550-dUTP, according to the manufacturer’s instructions. The (CA)_15_, (GA)_15_, (CGG)_10_ microsatellite probes were directly labeled with Cy3 during the synthesis, according to Kubat et al. (2008). In addition, since it contains the lowest 2n, telomeric (TTAGGG)_*n*_ sequence was also used as probe in *P. marylinae*. This probe was generated by PCR in the absence of a template according to Ijdo et al. (1991) and later labeled with ATTO550-dUTP with the Nick-Translation Mix Kit (Jena Bioscience, Jena, Germany). FISH experiments followed the methodology described in Yano et al. (2017). Metaphase chromosomes were treated with RNAse A (40 μg/ml) for 1.5 h at 37°C and the DNA denatured in 70% formamide/2× SSC at 72°C for 3.15 min. A hybridization mixture (2.5 ng/μL probes, 50% deionized formamide, 10% dextran sulfate) was then dropped on the slides, and the hybridization process was performed overnight at 37°C in a moist chamber. The first post-hybridization wash was performed with 1× SSC for 5 min at 65°C, followed by the second one performed with 4xSSC/Tween for 5 min, at room temperature. Chromosomes were then counterstained with DAPI, and the slides were mounted with an antifade solution (Vectashield from Vector Laboratories, Burlingame, CA).

### FISH for Whole Chromosome Painting

As *P. semifasciata* represents the only *Pyrrhulina* species that harbors an X_1_X_2_Y multiple sex system, a Y-chromosome probe, named PSEMI-Y, was previously prepared by microdissection, as described in (de Moraes et al., 2019) Male and female metaphases of *P. marilynae*, *Pyrrhulina* aff. *marilynae*, *Pyrrhulina* sp., *P. obermulleri*, *Pyrrhulina* cf. *laeta* were used for Zoo-FISH experiments with the PSEMI-Y probe, according to procedures described in Yano et al. (2017). The hybridization was performed for 72 h at 37°C in a moist chamber, with post-hybridization washes with 1xSSC for 5 min at 65°C, and in 4xSSC/Tween (RT). 10 μg of male-derived C_*0*_t-1 DNA from *P. semifasciata* was used as suppressor in each experiment. Chromosomes were stained with DAPI (1.2 μg/ml) and the slides were mounted with an antifade solution, as described above.

### Probes for Comparative Genomic Hybridization

The genomic DNAs (gDNAs) from male and female specimens of *P. marilynae*, *Pyrrhulina* aff. *marilynae*, *Pyrrhulina* sp., *P. obermulleri*, *Pyrrhulina* cf. *laeta*, *P. australis*, *Pyrrhulina* aff. *australis*, *P. brevis*, and *P. semifasciata* were extracted from liver tissue by the standard phenol-chloroform-isoamyl alcohol method (Sambrook and Russell, 2001). For intraspecific comparisons, the male-derived gDNAs of all species were labeled with ATTO550-dUTP and the female gDNAs with ATTO 488-dUTP, by nick translation (Jena Bioscience, Jena, Germany). The repetitive sequences were blocked using unlabeled C_*0*_t-1 DNA in all experiments, according to (Zwick et al., 1997). The final hybridization mixture for each slide (20 μL) was composed of male- and female-derived gDNAs (500 ng each), plus 25 μg of female-derived C_*0*_t-1 DNA from the respective species. The probe was ethanol-precipitated, and the dry pellets were mixed in a hybridization mixture containing 50% formamide, 2× SSC, 10% SDS, 10% dextran sulfate, and Denhardt´s buffer, pH 7.0.

For interspecific comparisons, the gDNA of male specimens of *P*. *australis* (Paus), *Pyrrhulina* aff. *australis* (Pafa), *P. semifasciata* (Psem), *P. brevis* (Pbre), *P. marilynae* (Pmar), *Pyrrhulina* aff. *marilynae* (Pafm), *Pyrrhulina* sp. (Psp), *P.obermulleri* (Pobe) and *Pyrrhulina* cf. *laeta* (Pcfl) were hybridized against metaphase chromosomes of *P. marilynae*. This species was selected since it harbors the lowest 2n = 32 until now register for the genus, coupled with a remarkable karyotype differentiation. For this purpose, male-derived gDNA of *P. marilynae* was labeled with ATTO 550-dUTP, while the gDNAs of the other species were labeled with ATTO 488-dUTP (*P. australis*, *Pyrrhulina* aff. *marilynae*, *P. brevis* and *P. obermulleri*) or ATTO 425-dUTP (*Pyrrhulina* aff. *australis*, *Pyrrhulina* sp., *P. semifasciata* and *Pyrrhulina* cf. *laeta*), both through nick translation (Jena Bioscience, Jena, Germany).

The interspecific comparisons were divided into a set of four slides. In the first slide, the final probe mixture was composed of 500 ng of male-derived gDNA plus 10 μg of male-derived C_0_t-1 DNA of each of the following species: *P. marilynae*, *P*. *australis*, and *Pyrrhulina* aff. *australis*. In the second slide, the final probe mixture was composed of 500 ng of male-derived gDNA plus 10 μg of male-derived C_*0*_t-1 DNA of each one of the following species: *P. marilynae*, *Pyrrhulina* aff. *marilynae* and *Pyrrhulina* sp. In the third slide, the final probe mixture was composed of 500 ng of male-derived gDNA plus 10 μg of male-derived C_*0*_t-1 DNA of each one of the following species: *P. marilynae*, *P. brevis*, and *P. semifasciata*. Finally, in the fourth slide, the final probe mixture was composed of 500 ng of male-derived gDNA plus and 10 μg of male-derived C_*0*_t-1 DNA of each one of the following species: *P. marilynae*, *P. obermulleri*, and *Pyrrhulina* cf. *laeta*. The chosen ratio of probe vs. C_*0*_t-1 DNA amount was based on previous experiments performed in our fish studies (de Moraes et al., 2019; Toma et al., 2019; Sassi et al., 2020). The CGH experiments followed the methodology described in Sember et al. (2018).

### Microscopy and Images Processing

To confirm the diploid number, karyotype structure and FISH results inat least 30 metaphase spreads were analyzed per individual. The microscopy images were captured using an Olympus BX50 epifluorescence microscope (Olympus Corporation, Ishikawa, Japan) coupled with a CoolSNAP camera, and the images were processed using Image-Pro Plus 4.1 Software (Media Cybernetics, Silver Spring, MD, United States). Final images were optimized and arranged using Adobe Photoshop, version CC 2020. Chromosomes were classified as metacentric (m), submetacentric (sm), subtelocentric (st), or acrocentric (a), according to their arm ratios (Levan, 1964). As the males and females results showed no differences, only male metaphases were represented in the figures.

## Results

### Karyotypes and Heterochromatin Distribution

**FIGURE 2:**
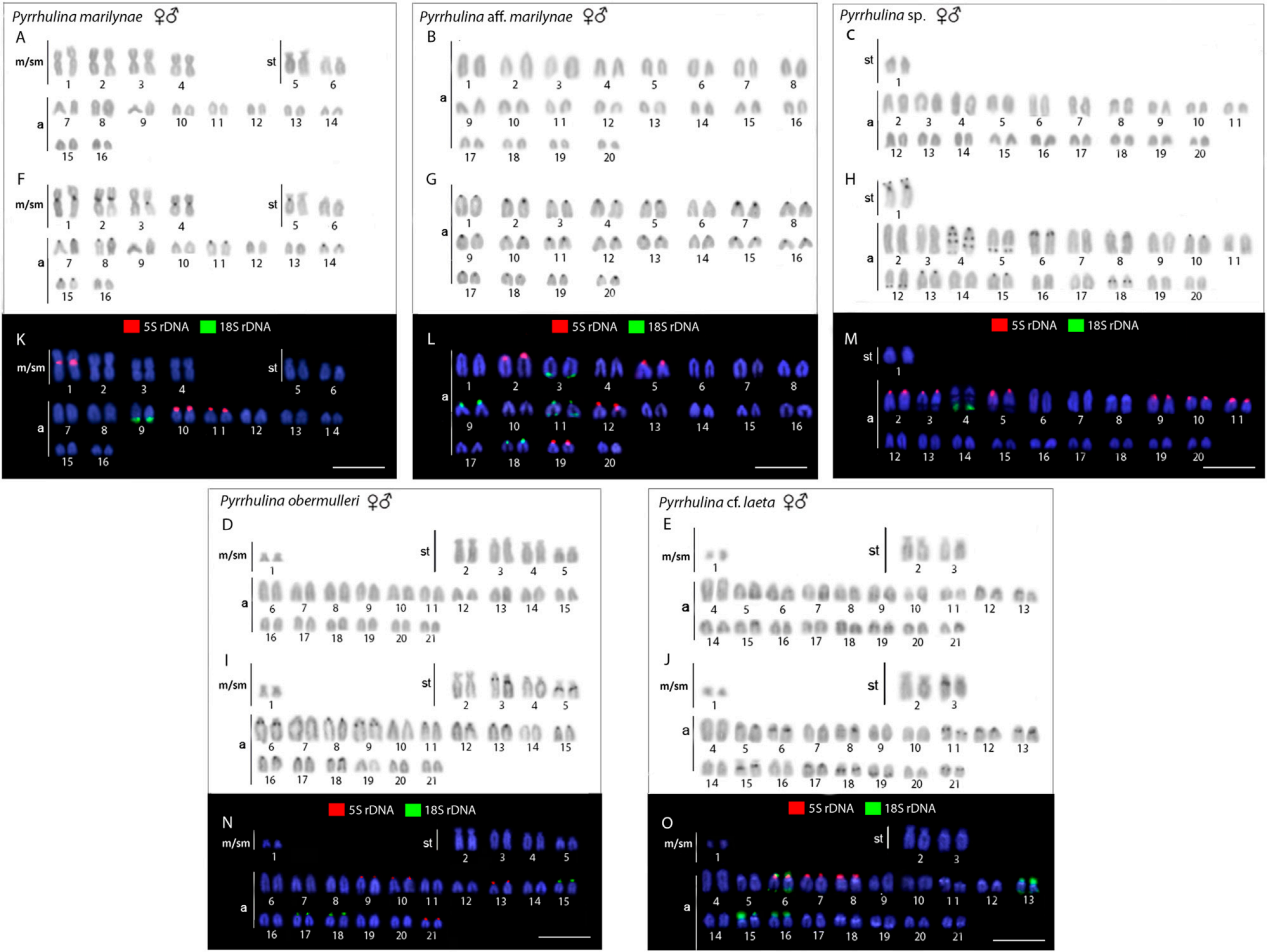
Male and female karyotypes of *Pyrrhulina marilynae* **(A, F, and K)**, *Pyrrhulina* aff. *marilynae* **(B, G and L)**, *Pyrrhulina* sp. **(C, H and M)**, *P. obermulleri* **(D, I and N)** and *Pyrrhulina* cf. *laeta* **(E, J and O)** arranged after Giemsa staining **(A-E)**, C-banding **(F-J)**, and dual-color *in situ* hybridization (FISH) with 18S (green) and 5S (red) ribosomal DNA probes **(K-O)**. Chromosomes were counterstained with 4′,6-diamidino-2-phenylindole (DAPI).Scale bar = 5 μm.

The diploid number ranged from 2n = 40 to 42 among the following four species: *Pyrrhulina* sp. (2n = 40; 2st+38a), *Pyrrhulina* aff. *marilynae* (2 = 40; 40a), *P. obermulleri* (2n = 42; 2m/sm+8st+32a) and *Pyrrhulina* cf. *laeta* (2n = 42; 2m/sm+4st+36a), the two latter also sharing a characteristic small metacentric/submetacentric pair. On the other hand, *P. marilynae* differed by presenting a very distinct karyotype composition (2n = 32; 8m/sm+4st+20a). These results represent the first cytogenetic data for the abovementioned species. The constitutive heterochromatin was distributed at the pericentromeric region of several chromosome pairs in *P. marilynae* and *Pyrrhulina* aff. *marilynae*. In its turn, *Pyrrhulina* sp., *P. obermulleri*, and *Pyrrhulina* cf. *laeta* presented a remarkable series of interstitial and pericentromeric C-bands, in addition to telomeric ones (Figure 2). In our sampling, we did not observe any karyotype differences between males and females.

### Chromosomal Mapping of Repetitive DNA Sequences

**FIGURE 3:**
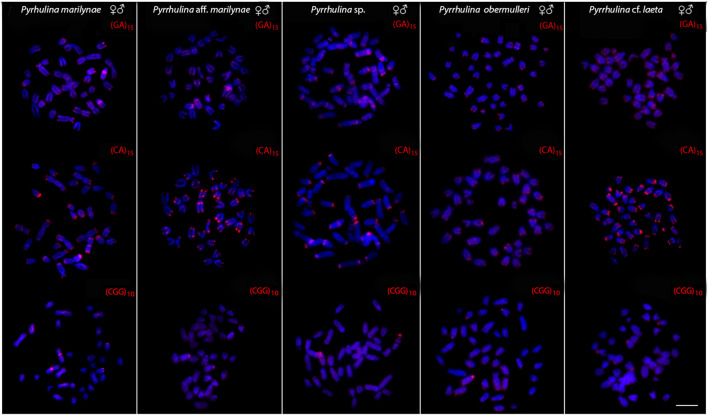
Male and female metaphase plates of *Pyrrhulina marilynae*; *Pyrrhulina* aff. *marilynae*; *Pyrrhulina* sp.; *P. obermulleri* and *Pyrrhulina* cf. *laeta* shows the general distribution of the microsatellites (GA)_15_, (CA)_15_ and (CGG)_10_ on chromosomes. Bar = 5 μm.

**FIGURE 4:**
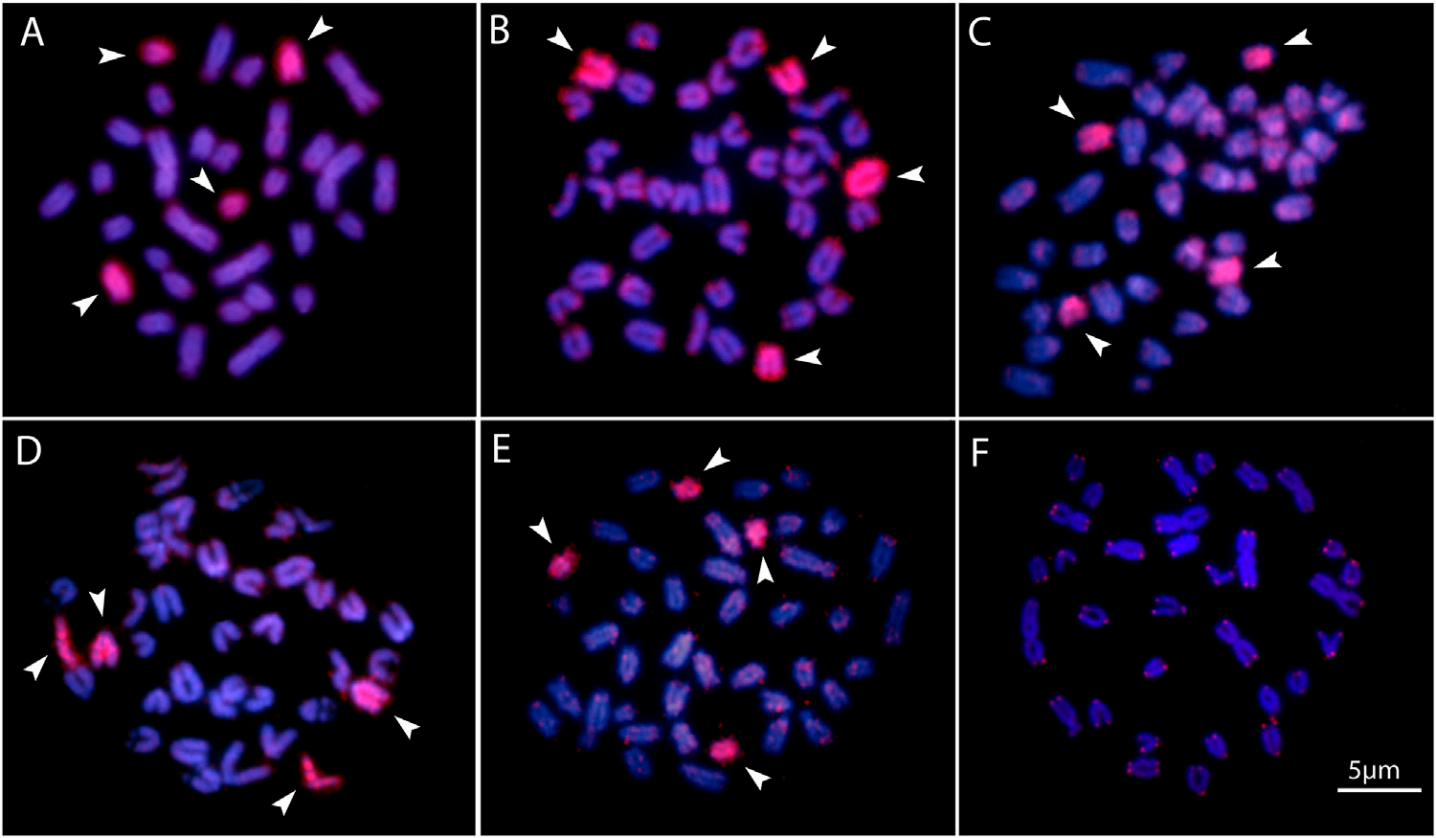
Zoo-FISH with the PSEMI-Y probeon male metaphase plates of *P. marilynae* **(A)**, *Pyrrhulina* aff. *marilynae* **(B)**, *Pyrrhulina* cf. *laeta* **(C)**, *Pyrrhulina* sp. **(D)**, and *P. obermulleri* **(F)** shows the distribution of the telomeric (TTAGGG)n repeats in *P. marilynae*. Bar = 5 μm.

All the five species differ by the distribution of the multigene rDNA families. *Pyrrhulina* sp. and *P. marilynae* were the only species with only one chromosome pair bearing 18S rDNA sites, found at the telomeric region of acrocentric pairs 4 and 9, respectively. Six to twelve centromeric or telomeric sites occur in the other three species, including bitelomeric sites in *Pyrrhulina* aff. *marilynae* (pair 11) and *Pyrrhulina* cf. *laeta* (pairs 6 and 13). As for the 5S rDNA, from six to twelve centromeric sites occured among species, including a syntenic condition for the 5S and 18S rDNA repeats in the chromosome pair 6 of *Pyrrhulina* cf. *laeta*, the same pair that displays bitelomeric 18S rDNA signals in this species (Figure 2). The distribution of the microsatellites (CA)_15_, (GA)_15_, and (CGG)_10_ does not differ significantly among species, having a preferential location in the centromeric and telomeric regions of the chromosomes, in addition to some interstitial sites. However, (CA)_15_ differs quantitatively, with a greater number of conspicuous sites compared to the other microsatellites, especially in *Pyrrhulina* aff. *marilynae* and *Pyrrhulina* cf. *laeta*. In the same way, (CGG)_10_ occurs in smaller amounts in the five species (Figure 3). The (TTAGGG)n repeats showed the expected hybridization signals on telomeres of *P. marylinae* (Figure 4F). Whole chromosome painting–WCP.

Two acrocentric chromosome pairs were entirely painted with the PSEMI-Y probe in *Pyrrhulina marilynae*, *P. obermulleri*, *Pyrrhulina* sp., *Pyrrhulina* aff. *marilynae* and *Pyrrhulina* cf. *laeta* (Figures 4A–E).

### Comparative Genomic Hybridization–CGH

**FIGURE 5:**
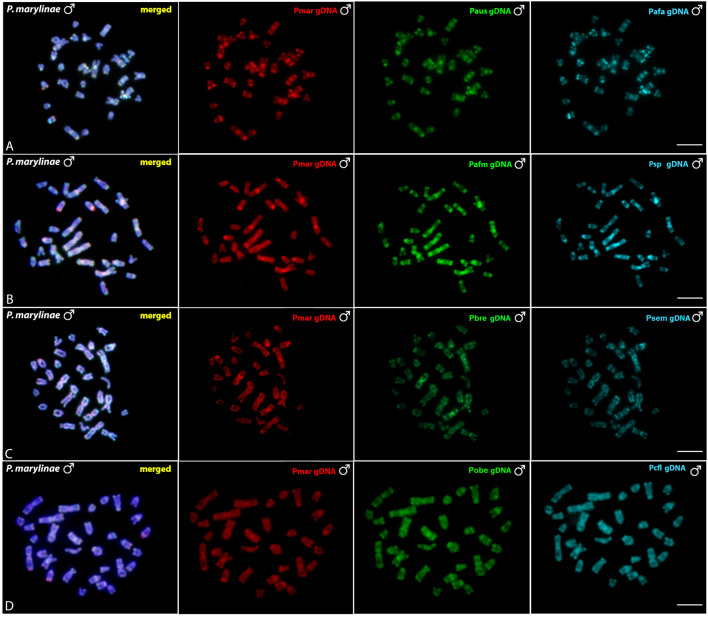
Comparative genomic hybridization (CGH) using male-derived genomic probes from *Pyrrhulina* species hybridized onto male chromosomes of *P. marilynae*. The common genomic regions are depicted in the 1^st^ column in each line representing the experiments A-D. Hybridization between the gDNA of *P. marilynae* (Pmar), *P. australis* (Paus) and *Pyrrhulina* aff. *australis* (Pafa) **(A)**; *P. marilynae*(Pmar), *Pyrrhulina* aff. *marilynae* (Pafm) and *Pyrrhulina* sp. (Psp) **(B)**; *P. marilynae*(Pmar), *P. brevis* (Pbre) and *P. semifasciata* (Psem) **(C)**; *P. marilynae* (Pmar), *P. obermulleri* (Pobe) and *Pyrrhulina* cf. *laeta* (Pcfl) **(D)**. Bar = 5 μm.

The interespecific genomic comparison among *Pyrrhulina marilynae* and other *Pyrrhulina* species (*P. semifasciata*, *P*. *australis*, *P. brevis*, *P. obermulleri*, *Pyrrhulina* aff. *australis*, *Pyrrhulina* sp., *Pyrrhulina* aff. *marilynae*, *Pyrrhulina* cf. *laeta*) revealed a high level of DNA compartmentalization, within all species presenting a distinct composition of repetitive DNA sequences and specific signals. However, *P. marilynae* shows more evident species-specific arrangements when compared to the other species. (Figure 5). Intraspecific genomic hybridization between males and females did not show any clustering for sex-specific sequences in all species (data not shown).

## Discussion

Overall, two main evolutionary trends are proposed for the karyotypic evolution of the Lebiasinidae: 1) the conservation of a plesiomorphic karyotype in the subfamily Lebiasininae, with 2n = 36 bi-armed chromosomes and, 2) high variations in diploid numbers and karyotypic structures in the subfamily Pyrrhulininae, with the predominance of acrocentric chromosomes (Sassi et al., 2020). It is noteworthy that the karyotypic structure of Lebiasininae, 2n = 36 biarmed chromosomes, is similar to that found in the sister family Ctenoluciidae (de Souza e Sousa et al., 2017; Sassi et al., 2019; de Souza e Sousa et al., 2021). Therefore, in this scenario, the majority of the acrocentric chromosomes found in the species of the Pyrrhulininae are probably derived from rearrangements such as centric fissions (Sassi et al., 2020). However, unlike other *Pyrrhulina* species, *P. marilynae* has the smallest 2n identified in the genus so far, 2n = 32, including four typical meta/submetacentric pairs. Some exceptions within the subfamily showed secondary fusion events of acrocentric chromosomes giving rise to metacentric chromosomes, reducing the diploid number as observed in *Nannostomus unifasciatus* (Sember et al., 2020). Biarmed chromosomes could also represent remnants of the ancestral karyotype condition that were maintained during the evolutionary processes. However, no ITS was found in any chromosome of *P. marilynae*, but such a scenario does not exclude the hypothesis of fusion, given that telomeric regions can be lost after the rearrangement occurs (Bolzán, 2017). Thus, to corroborate such hypotheses and to determine whether the evolutionary trajectory of karyotype change in *Pyrrhulina* is directed mainly towards centric fusions or fissions, cytogenetic data should be discussed in a larger phylogenetic framework of interspecific and intergeneric relationships of Lebiasinidae.

**FIGURE 6:**
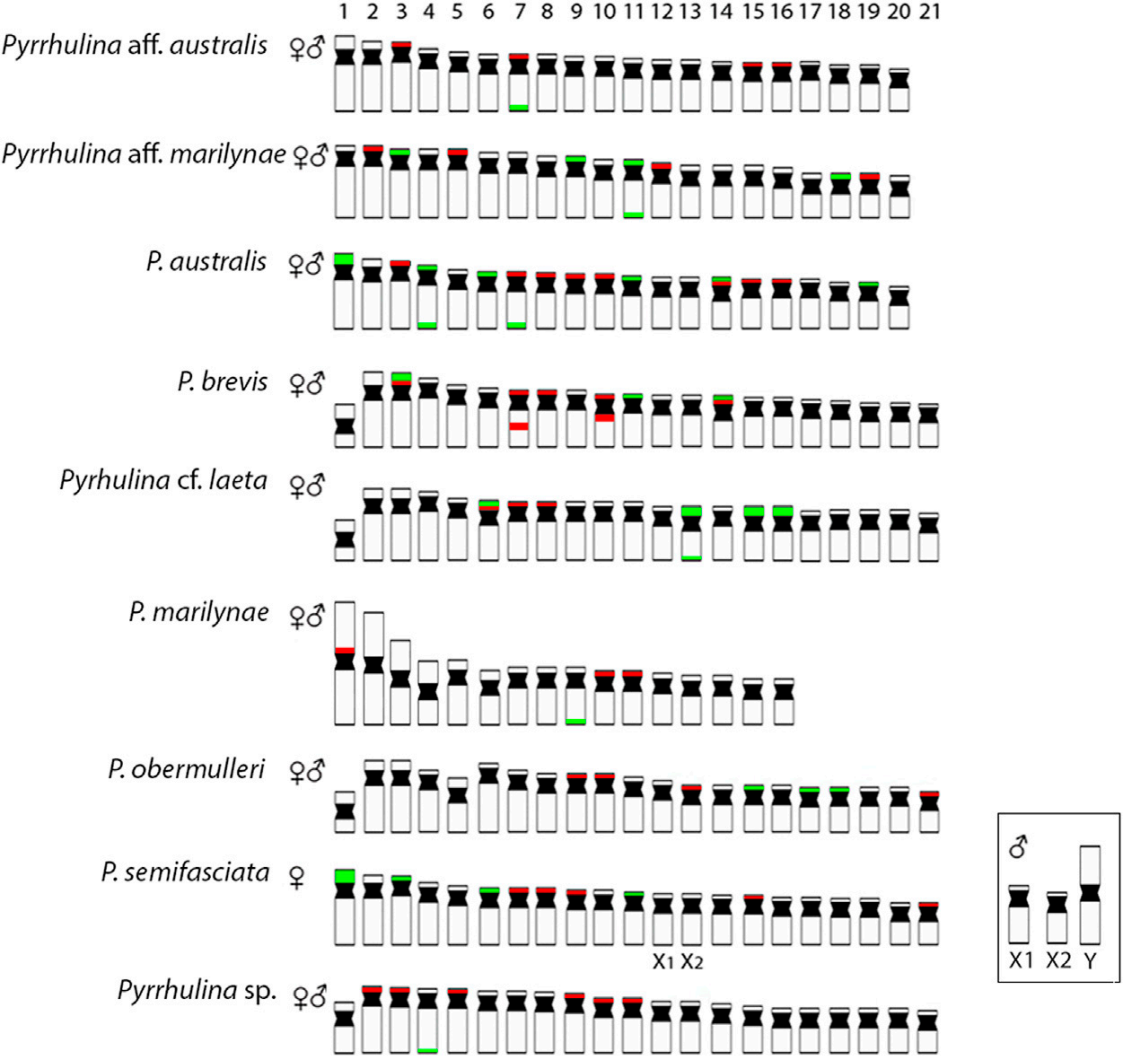
Representative idiograms of *Pyrrhulina* species showing the distribution of the 18S (green) and 5S rDNA (red) sites on chromosomes, based on the present study and some other previous data (de Moraes et al., 2017; de Moraes et al., 2019). Bar = 5 μm.

CGH procedures have greatly assisted cytogenetic studies (Symonová et al., 2013; Cioffi et al., 2017; Cioffi et al.,2019), as among all *Pyrrhulina* studied so far. In fact, despite showing close genomic similarities, the species also show considerable divergences, in addition to species-specific repetitive DNA and C-band patterns, thus helping to understand their differential evolutionary paths, considering the taxonomic problems still pending in this fish group. In addition, multiple and syntenic ribosomal sites are not frequently observed among fishes, but these chromosomal features are very informative cytotaxonomic markers regarding Pyrrhulininae species. Comparatively, they occur more frequently among *Pyrrhulina* than in other species of this subfamily (de Moraes et al., 2017; de Moraes et al.,2019; Sassi et al., 2019; Sassi et al.,2020; Toma et al., 2019; Sember et al., 2020). Like *Pyrrhulina* aff. *australis* (de Moraes et al., 2017), *Pyrrhulina* sp., and *P. marilynae* present multiple 5S rDNA sites and only one 18S rDNA site, thus differentiating them from *Pyrrhulina* aff. *marilynae*, *P. obermulleri*, and *Pyrrhulina* cf. *laeta*, as well as from some other *Pyrrhulina* species (de Moraes et al., 2017; de Moraes et al.,2019), which have multiple 5S and 18S rDNA sites. Furthermore, the syntenic condition for the 18S/5S rDNAs in *Pyrrhulina* cf. *laeta* is shared with *P. brevis* and *P*. *australis*, indicating a high rDNA diversity. (Figure 6). In its turn, the 18S rDNA clusters are distributed on distal chromosome positions for all investigated *Pyrrhulina* species (de Moraes et al., 2017; de Moraes et al.,2019; this study), as also occur among *Copeina* (Toma et al., 2019), *Lebiasina* (Sassi et al., 2019), and *Nannostomus* (Sember et al., 2020), so as in the species of the sister family, Ctenoluciidae (de Souza e Sousa et al., 2017; de Souza e Sousa et al., 2021).

Microsatellite distribution patterns have significantly contributed to evolutionary studies in fish species, especially regarding sex chromosome differentiation (Kubat et al., 2008; Cioffi et al., 2012; Terencio et al., 2012; Kejnovský et al., 2013; Poltronieri et al., 2014; Yano et al., 2014; de Freitas et al., 2018). Among the five *Pyrrhulina* species now investigated, as well as in other previous analyzed ones (de Moraes et al., 2017; de Moraes et al.,2019), the distribution of the microsatellites did not significantly differ among them, although the (CA)_15_ repeats present a greater number of more conspicuous sites than the other microsatellites, especially in *Pyrrhulina* aff. *marilynae* and *Pyrrhulina* cf. *laeta*. It is noteworthy that microsatellites have a preferential location in the telomeric and centromeric regions of fish chromosomes (Cioffi and Bertollo, 2012), as occur with the (CA)_15_ and (GA)_15_ motifs in *Pyrrhulina*, despite some interstitial and pericentromeric signs in *Pyrrhulina* cf. *laeta*, *P. marilynae*, *Pyrrhulina* aff. *marilynae* and *Pyrrhulina* sp., thus differentiating these species from others previously studied (de Moraes et al., 2017; de Moraes et al.,2019). Furthermore, it is also frequent that microsatellites and other repetitive sequences occur in the association among fish (Cioffi and Bertollo, 2012), such as in *Hepsetus odoe* (Carvalho et al., 2017), *Lebiasina bimaculata* (Sassi et al., 2019), and *Silurichthys phaiosoma* (Ditcharoen et al., 2020), for example. This is the scenario that also occurs in *Pyrrhulina* sp., in which the (CGG)_10_ microsatellite located in the telomeric region of pair 4 shares the same chromosomal region with 18S rDNA repeats.

Fish, besides presenting high diversity in morphological and genetic characteristics, also have a variety of sex chromosome systems (Sember et al., 2021). About nine differentiated systems, involving the XX/XY and ZZ/ZW sex chromosomes and their variations, have been identified among species, including several Neotropical ones (Sember et al., 2021). It is noteworthy that among the multiple systems, the ♀X_1_X_1_X_2_X_2_/♂X_1_X_2_Y is the most prevalent one, and commonly originated by centric or tandem fusions of the ancestral Y with an autosomal member of the karyotype, giving rise to neo-Y chromosomes, as identified in a variety of fish species (Sember et al., 2021). That includes *P. semifasciata*, the only Lebiasinidae representative highlighting heteromorphic sex chromosomes so far (de Moraes et al., 2019), in addition to a putative ZZ/ZW sex system present *in Lebiasina bimaculata* (Sassi et al., 2019). Although our intraspecific CGH results in the current analyzed species did not reveal any sex-specific differentiated region, our WCP experiment with the Y-derived probe of *P. semifasciata* entirely painted two acrocentric pairs, suggesting that putative proto-XY chromosomes may occur in these species. Thus, it supports our previous hypothesis on the origin of the *P. semifasciata* sex chromosome system through centric fusion between the non-homologous acrocentric, giving rise to the large metacentric Y chromosome. That can be considered as an apomorphy of this species when compared to others of the genus. Furthermore, the experiments also showed that although the karyotype of *P. marilynae* has large metacentric chromosomes, these do not correspond to the heteromorphic sex chromosome of *P. semifasciata* (Figure 4).

## Conclusion

Our data advances the understanding of evolutionary trends of the Lebiasinidae, particularly concerning *Pyrrhulina*. Karyotypes with 2n = 40–42, with the predominance of mono-armed chromosomes, are more frequent among its species, except for *P. marilynae*, which has a smaller diploid number (2n = 32), and several atypical biarmed chromosomes, a characteristic that differentiates this species from the others analyzed in the genus. However, we cannot rule out the hypothesis that this karyotypic reduction (2n = 32) may have been generated by secondary fusions that allowed the formation of the four meta/submetacentric pairs identified in *P. marilynae*. The present data also highlighted the putative proto-XY chromosomes that may occur in these species and support the occurrence, through centric fusion, of the multiple sex chromosome system of *P. semifasciata* as an independent evolutionary event of this Lebiasinidae species. Our results highlight the importance of chromosomal data as valuable markers for understanding the evolutionary relationships among Lebiasinidae species.

## Data Availability

The original contributions presented in the study are included in the article/Supplementary Material, further inquiries can be directed to the corresponding author.
